# Artificial Intelligence–Powered Spatial Analysis of Immune Phenotypes in Resected Pancreatic Cancer

**DOI:** 10.1001/jamasurg.2025.1999

**Published:** 2025-06-25

**Authors:** Hyemin Kim, Jin Ho Choi, Yoojoo Lim, So Jeong Yoon, Kee-Taek Jang, Chan-Young Ock, Young Hoon Choi, Cheolyong Joe, Sanghoon Song, Jimin Moon, Heon Song, Sergio Pereira, Seungeun Lee, Sujin Park, Kyunga Kim, Se-Hoon Lee, Hongbeom Kim, Sang Hyun Shin, Jin Seok Heo, Kwang Hyuck Lee, Kyu Taek Lee, Jong Kyun Lee, In Woong Han, Joo Kyung Park

**Affiliations:** 1 Department of Medicine, Samsung Medical Center, Sungkyunkwan University School of Medicine, Seoul, Republic of Korea; 2 Department of Internal Medicine, Seoul National University Hospital, Seoul National University College of Medicine, Seoul, Korea; 3 Lunit, Seoul, Republic of Korea; 4 Division of Hepatobiliary-Pancreatic Surgery, Department of Surgery, Samsung Medical Center, Sungkyunkwan University School of Medicine, Seoul, Republic of Korea; 5 Department of Pathology and Translational Genomics, Samsung Medical Center, Sungkyunkwan University School of Medicine, Seoul, Republic of Korea; 6 Department of Health Sciences and Technology, Samsung Advanced Institute of Health Sciences and Technology, Sungkyunkwan University, Seoul, Republic of Korea; 7 Department of Pathology, Shinwon Medical Foundation, Gwangmyeong, Republic of Korea; 8 Biomedical Statistics Center, Research Institute for Future Medicine, Samsung Medical Center, Seoul, Republic of Korea; 9 Department of Clinical Research Design & Evaluation, Samsung Advanced Institute of Health Sciences and Technology, Sungkyunkwan University, Seoul, Republic of Korea

## Abstract

**Importance:**

Although tumor-infiltrating lymphocytes (TILs) have been implicated as prognostic biomarkers across various malignancies, the clinical application remains challenging. This study evaluated the applicability of artificial intelligence (AI)–powered spatial mapping of TIL density for prognostic assessment in resected pancreatic ductal adenocarcinoma (PDAC).

**Objective:**

To evaluate the prognostic significance of AI-powered spatial TIL analysis in resected PDAC and its clinical applicability.

**Design, Setting, and Participants:**

This cohort study included patients with PDAC who underwent up-front R0 resection at a tertiary referral center between January 2017 and December 2020. Whole-slide images of retrospectively enrolled patients with PDAC and up-front R0 resection were analyzed. An AI-powered whole-slide image analyzer was used for spatial TIL quantification, segmentation of tumor and stroma, and immune phenotype classification as immune-inflamed phenotype, immune-excluded phenotype, or immune-desert phenotype. Study data were analyzed from January 2017 to August 2023.

**Exposure:**

Use of AI-powered spatial analysis of the tumor microenvironment in resected PDACs.

**Main Outcomes and Measures:**

Tumor microenvironment–related risk factors and their associations with overall survival (OS) and recurrence-free survival (RFS) outcomes were identified.

**Results:**

Among 304 patients, the mean (SD) age was 66.8 (9.4) years with 171 male patients (56.3%), and preoperative clinical stages I and II were represented by 54.3% patients (165 of 304) and 45.7% patients (139 of 304), respectively. The TILs in the tumor microenvironment were predominantly concentrated in the stroma, and the median intratumoral TIL and stromal TIL densities were 100.64/mm^2^ (IQR, 53.25-121.39/mm^2^) and 734.88/mm^2^ (IQR, 443.10-911.16/mm^2^), respectively. Overall, 9.9% of tumors (30 of 304) were immune inflamed, 85.2% (259 of 304) were immune excluded, and 4.9% (15 of 304) were immune desert. The immune-inflamed phenotype was associated with the most prolonged OS (median not reached; *P* < .001) and RFS (median not reached; *P* = .001), followed by immune-excluded phenotype and immune-desert phenotype. High intratumoral TIL density was associated with longer OS (median, 52.47 months; 95% CI, 41.98-62.96; *P* = .004) and RFS (median, 21.67 months; 95% CI, 14.43-28.91; *P* = .02). A combined analysis of the pathologic stage with immune phenotype predicted better survival of stage II PDAC stratified as immune-inflamed phenotype than stage I PDAC stratified as non–immune-inflamed phenotype.

**Conclusions and Relevance:**

Results of this cohort study suggest that the use of AI has markedly condensed the labor-intensive process of TIL assessment, potentially rendering the process more feasible and practical in clinical application. Importantly, the IP may be one of the most important prognostic biomarkers in resected PDACs.

## Key Points

**Question:**

Can artificial intelligence (AI)–powered spatial analysis of tumor-infiltrating lymphocytes (TILs) provide prognostic value in resected pancreatic ductal adenocarcinoma (PDAC)?

**Findings:**

In this cohort study including 304 patients with resected PDAC, AI-powered immune phenotype classification was a critical independent factor for predicting survival outcomes, complementing the existing pathologic staging system. The immune-inflamed phenotype had the longest survival, whereas the immune-desert phenotype had the worst; high intratumoral TIL density correlated with improved outcomes.

**Meaning:**

Study results suggest that AI-powered spatial TIL analysis may serve as a practical and scalable biomarker for prognostic stratification in PDAC, supporting its practical integration alongside conventional staging.

## Teaser

This cohort study investigates if artificial intelligence–powered spatial analysis of tumor-infiltrating lymphocytes can provide prognostic value in resected pancreatic ductal adenocarcinoma.

## Introduction

Pancreatic ductal adenocarcinoma (PDAC) is a deadly disease without strong prognostic predictors and with limited effectiveness of systemic therapies.^1^ A typical histological feature of PDAC is a desmoplastic reaction to the tumor, which is considered to lead to poor prognosis by forming a physical barrier for the delivery of chemotherapeutic drugs and infiltration of immune cells as well as by preventing appropriate vascularization.^2,3,4^ However, the role of the immune environment in PDAC remains to be revealed. The exact immune landscape and underlying mechanisms are being actively investigated, but the specific immune landscape and mechanisms involved in PDAC and the adjacent tumor microenvironment (TME) have yet to be elucidated. Recently, spatial analysis of immune components in PDAC has provided greater insight into the role of the TME.^3,5^ However, there is still an unmet need to predict the prognosis of PDAC patients with TME-based considerations.

The degree of tumor-infiltrating lymphocyte (TIL) infiltration in tumors and the adjacent TME has been suggested to be a prognostic factor in several cancer types, including PDAC, but it has rarely been used in actual clinical practice due to its high barrier of evaluation; furthermore, the standard evaluation methods and resulting cutoff values for assessing prognosis are not well established.^6,7,8,9,10^ Interestingly, the degree of intratumoral TIL infiltration has been suggested to be a prognostic factor in PDAC, and a recent meta-analysis^5^ revealed that high infiltration of CD8^+^ lymphocytes, CD3^+^ T cells, and CD4^+^ lymphocytes was correlated with improved survival outcomes. High expression of FoxP3^+^ lymphocytes was associated with poor survival and the spatial distribution of cytotoxic T cells near cancer cells were correlated with increased overall survival (OS).^5,6,7^ Unfortunately, despite these promising results, identifying TIL density is a very labor-intensive and subjective task for pathologists, and it seems unfeasible for humans to accurately and consistently assess TILs without interexaminer or intraexaminer variance.

Lunit SCOPE IO is an artificial intelligence (AI)–powered spatial TIL analyzer for all cancer types that can analyze the composition of the TME from hematoxylin-eosin (H&E)–stained whole-slide images (WSIs).^11^ It can recognize and segment the cancer area and cancer stroma within a WSI and identify TILs to determine spatial TIL densities and classify immune phenotypes (IPs). This study was designed to (1) classify IPs in resected PDAC and (2) determine the clinical implication of IPs as a prognostic factor in resected PDAC.

## Methods

### Study Population and AI-Powered IP Analysis

This study complies with the Declaration of Helsinki and was performed according to the ethics committee approval of the institutional review board at Samsung Medical Center, Seoul, Korea (No. SMC-2022-03-136) with written informed consent. In this retrospective study, patients with PDAC who underwent upfront surgery at Samsung Medical Center (Seoul, Korea) were eligible for enrollment between January 2017 and December 2020. Patients for whom R0 resection was not achievable were excluded from the study. Patients with an inadequate follow-up period or other complications leading to indeterminate prognosis were excluded (eFigure 1A in Supplement 1). All participants were Korean. Lunit SCOPE IO (Lunit Inc) was used for the AI-powered WSI analysis of the H&E-stained surgical specimen (eFigure 1B in Supplement 1). The spatial TIL density classified IP into 3 types: immune-inflamed phenotype (IIP), immune-excluded phenotype (IEP), and immune-desert phenotype (IDP).^12^ We further analyzed subgroup’s immune cell composition and immune cytotoxic activity. Detailed methods are described in the eMethods in Supplement 1. This study followed the Strengthening the Reporting of Observational Studies in Epidemiology (STROBE) reporting guidelines.

### Statistical Analysis

Group differences in continuous variables were assessed using nonparametric tests. Recurrence-free survival (RFS) and overall survival (OS) were estimated via Kaplan-Meier analysis. Cox proportional hazards models adjusted for confounders, with sensitivity analyses for potential proportional hazards violations. Statistical significance was set at a 2-sided *P* value < .05. Analyses were performed using R, version 4.2.2 (R Project for Statistical Computing) and SPSS, version 23.0 (IBM Corp). Study data were analyzed from January 2017 to August 2023. Detailed statistical methods are in the eMethods in Supplement 1.

## Results

### Dataset

Among the 304 patients, the mean (SD) age was 66.8 (9.4) years; 133 patients (43.7%) were female, and 171 patients (56.3%) were male (Table 1). The most frequently observed clinical stage was stage I (54.3% [165 of 304]), followed by stage II (45.7% [139 of 304]). The postoperative pathologic stages were stage II (45.4% [138 of 304]), stage I (42.1% [128 of 304]), and stage III (9.2% [38 of 304]). The median (IQR) follow-up time was 35.0 (17.4-46.8) months, and 188 clinical recurrences and 190 death events were observed during the study period. Among the deceased patients, 27 died without experiencing a recurrence. The estimated median RFS was 15.8 months (95% CI, 11.29-20.41 months) and the estimated median OS was 35.87 months (95% CI, 31.73-40.01 months). Baseline clinical and pathological characteristics were comparable among the 3 IP groups (eTable 1 in Supplement 1).

**Table 1.** Patient Characteristics

Characteristic	No. (%)
Study patients, No.	304
Age, mean (SD), y	66.83 (9.38)
Sex	
Female	133 (43.7)
Male	171 (56.3)
Operation type	
Pancreaticoduodenectomy	167 (55.0)
Left-sided pancreatectomy	122 (40.1)
Total pancreatectomy	15 (4.9)
Clinical stage (AJCC 8th), preoperative	
I	165 (54.3)
II	139 (45.7)
Pathologic stage (AJCC 8th), postoperative	
I	128 (42.1)
II	138 (45.4)
III	38 (9.2)
Pathologic T stage	
pT1	64 (21.1)
pT2	212 (69.7)
pT3	28 (9.2)
Pathologic N stage	
N0	138 (45.4)
N1	127 (41.8)
N2	39 (12.8)
Perineural invasion	
Negative	47 (15.5)
Positive	252 (82.9)
NA	5 (1.6)
Lymphovascular invasion	
Negative	143 (47.0)
Positive	156 (51.3)
NA	5 (1.6)
Differentiation	
Well/moderately differentiated	230 (75.7)
Poorly/undifferentiated	69 (22.7)
NA	5 (1.6)
Adjuvant therapy	
Yes	238 (78.3)
No	66 (21.7)
Recurrence	188 (61.8)
Death	190 (62.5)
Follow-up period, median (IQR), mo	35.01 (17.37-46.76)

Abbreviations: AJCC, American Joint Committee on Cancer; NA, not available.

### Distribution of IPs, Spatial TIL Densities, and TIL Subsets

**Figure 1.**
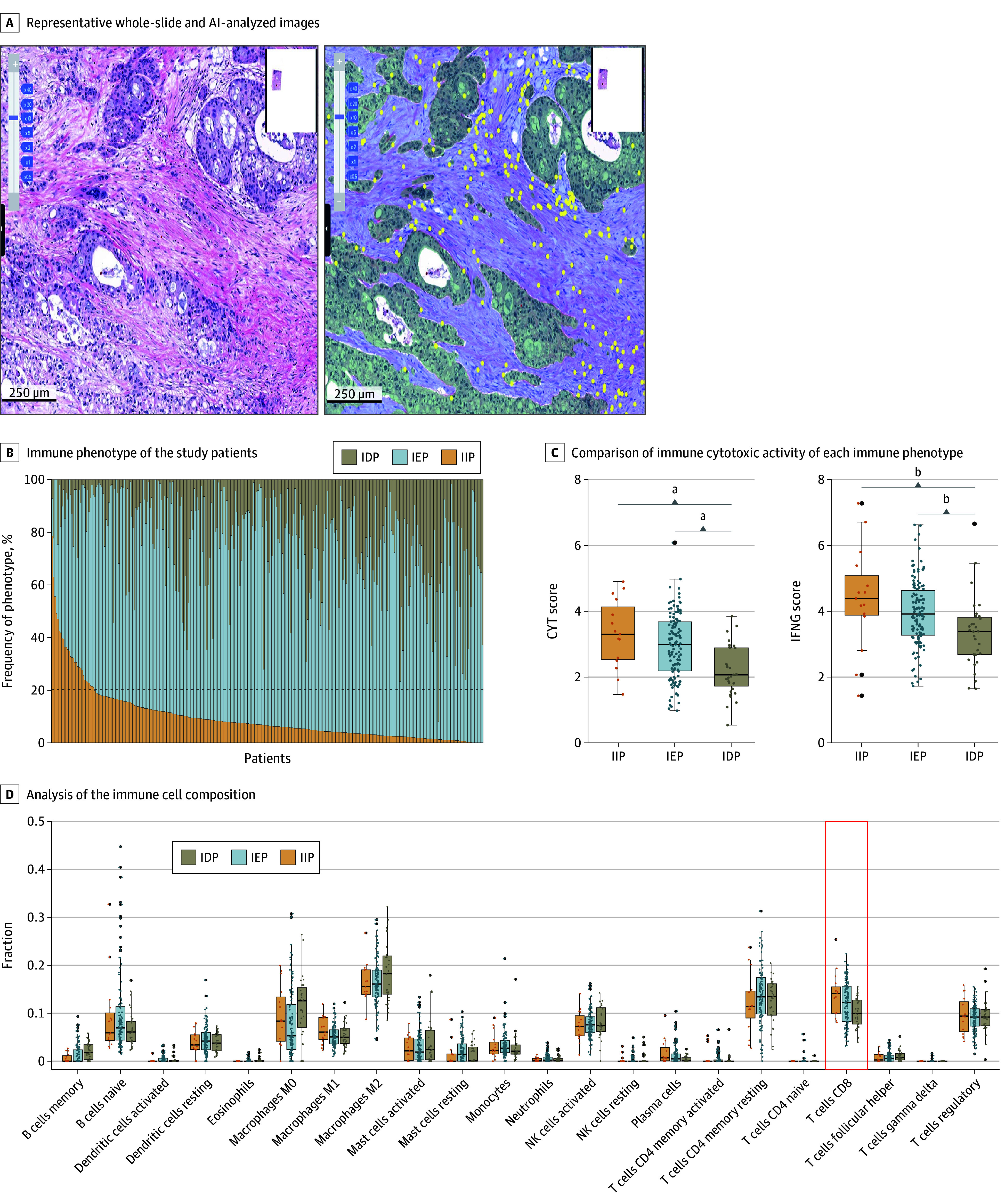
Distribution of Immune Phenotypes and Composition of Tumor-Infiltrating Lymphocytes A, Representative image of the original hematoxylin-eosin–stained whole-slide image (left) and Lunit SCOPE IO-inferenced image (right, tumor area, green; stroma area, blue; lymphocytes, yellow). B, Landscape of the immune phenotype of the study patients. C, CIBERSORTx (Stanford University), a machine learning method to infer cell type–specific gene expression profiles without physical cell isolation, analysis of the immune cell composition using The Cancer Genome Atlas (TCGA) Pancreatic Adenocarcinoma (PAAD) database. D, Comparison of the immune cytotoxic activity of each immune phenotype by cytolytic activity (CYT) score (left) and interferon-γ (IFNG) score (right) using the TCGA-PAAD database. Whiskers extend from the box to the minimum and maximum values within 1.5 × IQR from Q1 and Q3. Any point that falls outside the whiskers is considered an outlier. IDP indicates immune-desert phenotype; IEP, immune-excluded phenotype; IIP, immune-inflamed phenotype.

TILs were predominantly localized in the cancer stroma compartment (median intratumoral TIL density, 100.64/mm^2^ [IQR, 53.25-121.39/mm^2^]) rather than the cancer area compartment (median stromal TIL density, 734.88/mm^2^ [IQR, 443.10-911.16/mm^2^]). Representative images are shown in Figure 1A and the IP distribution in Figure 1B. Among 304 patients, IEP was the most common phenotype (85.2% [259 of 304]), followed by IIP (9.9% [30 of 304]) and IDP (4.9% [15 of 304]). The hazard ratio (HR) for RFS using the inflamed score as a continuous variable was 0.98 (95% CI, 0.97-0.99; *P* = .009). The optimal cutoff of the IS for identifying RFS in the 2 groups according to the lowest *P* value was approximately 20%. Notably, this threshold aligns with the tumor-agnostic IS cutoff for determining IIP independent of this study.

To investigate further insight into the subtypes of lymphocytes, we used The Cancer Genome Atlas (TCGA) Pancreatic Adenocarcinoma (PAAD) dataset (n = 172), and the median densities of intratumoral TILs and stromal TILs were 74.8/mm^2^ (IQR, 47.9-114.7/mm^2^) and 439.2/mm^2^ (IQR, 276.9-745.5/mm^2^), respectively. Among the participants, 8.7% (15 of 172) had IIP, 73.3% (126 of 172) had IEP, and 18.0% (31 of 172) had IDP. CIBERSORTx (Stanford University), a machine learning method to infer cell type–specific gene expression profiles without physical cell isolation, analysis of 22 immune cell types revealed a varied distribution of immune cell subsets based on the IP, as shown in Figure 1C. Notably, the fraction of CD8+ T cells (mean [SD], 0.14 [0.05] for IIP; 0.12 [0.04] for IEP; 0.10 [0.03] for IDP; *P* = .02) exhibited significantly different distributions compared with those in the IP. Additionally, immune cytotoxic activity tended to increase in the IIP group, followed by the IEP and IDP groups, as indicated by both the cytolytic activity score and interferon-γ expression being substantially more significant in the IIP group and lowest in the IDP group, as depicted in Figure 1D.

The distributions of IP and intratumoral TIL or stromal TIL densities according to clinicopathologic features are summarized in eTable 2 in Supplement 1. The intratumoral TIL density was significantly greater in patients with IIP (mean (SD), 286.04/mm^2^ [132.90/mm^2^] for IIP; 81.11/mm^2 ^[40.48/mm^2^] for IEP; 67.08/mm^2^ [38.90/mm^2^] for IDP *P* < .001), and the stromal TIL density was also significantly different (mean [SD], 1069.00/mm^2^ [541.34/mm^2^] for IIP; 723.99/mm^2^ [390.44/mm^2^] for IEP; 254.73/mm^2^ [87.28/mm^2^] for IDP; *P* < .001) according to the IP. The stromal TIL density was significantly greater in younger patients (mean [SD] stromal TIL density, 787.31/mm^2^ [419.66/mm^2^] in patients <67 years vs 688.32/mm^2^ [425.90/mm^2^] in patients ≥67 years; *P* = .04). Moreover, neither the IP nor the intratumoral TIL/stromal TIL density was significantly associated with other clinical factors.

### IPs and Intratumoral TILs as Predictors of Prognosis in Patients With Resected PDAC

**Figure 2.**
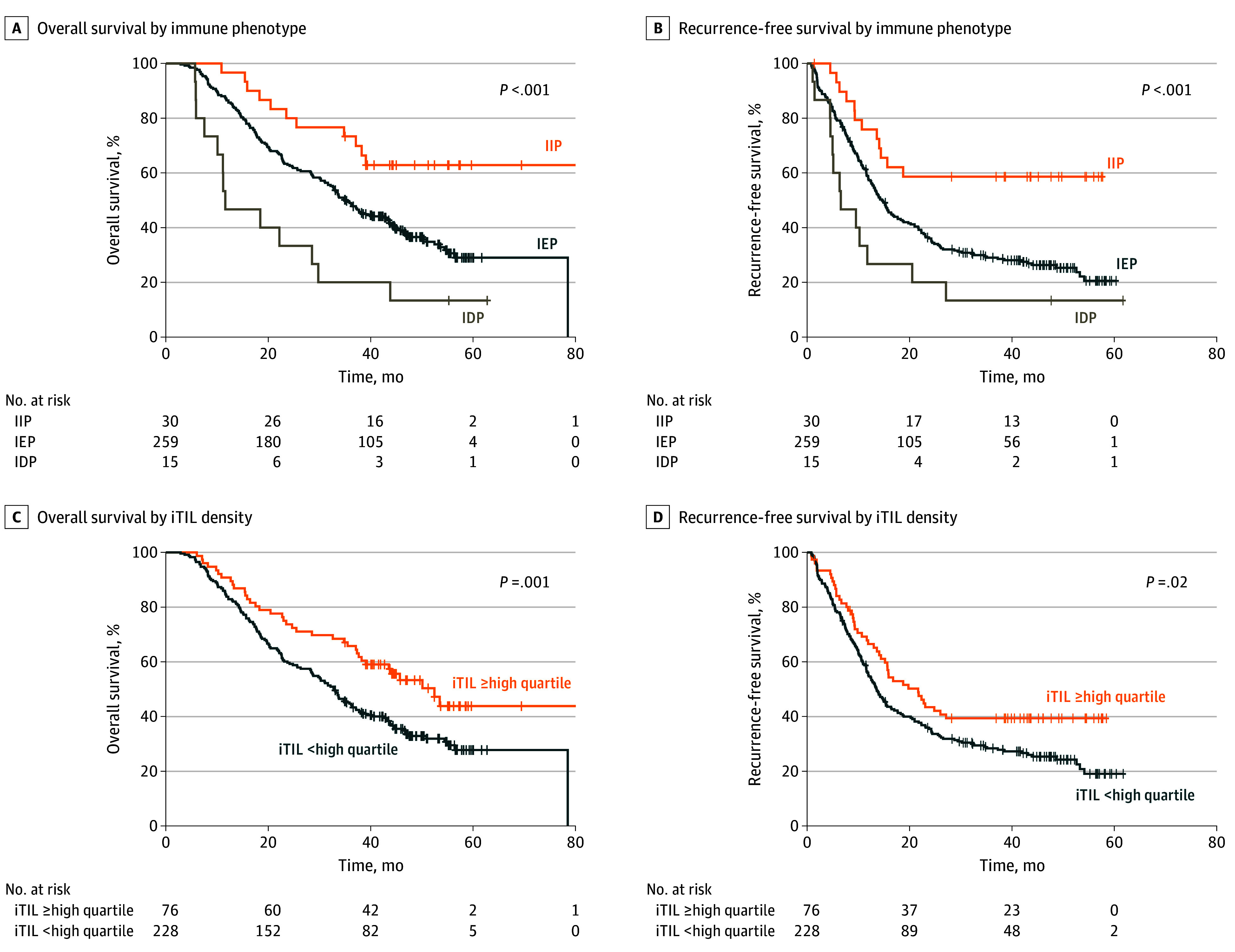
Kaplan-Meier Analysis for the Comparison of Survival Outcomes in the Study Patients A, Overall survival according to immune phenotype. The hazard ratio (HR) for immune-excluded phenotype (IEP) was 2.28 (95% CI, 1.23-4.21), and for immune-desert phenotype (IDP), HR was 5.20 (95% CI, 2.23-11.65). B, Recurrence-free survival according to the immune phenotype. For IEP, the HR was 2.42 (95% CI, 1.35-4.34), and for IDP, HR was 4.18 (95% CI, 1.91-9.19). C, Overall survival according to the intratumoral tumor-infiltrating lymphocyte (iTIL) density. The HR was 0.59 (95% CI, 0.41-0.85). D, Recurrence-free survival according to the iTIL density. The HR was 0.68 (95% CI, 0.49-0.94). IIP indicates immune-inflamed phenotype.

We analyzed survival outcomes in 304 patients based on pathological stage and spatial TIL distribution. The OS and RFS of the patients were significantly different according to the IPs (Figure 2A and B). IIP was associated with the most prolonged OS (median not reached) and RFS (median not reached), followed by IEP (median, 35.11 months; 95% CI, 31.33-38.90 months for OS; 14.63 months; 95% CI, 12.52-16.75 months for RFS) and IDP (median, 11.6 months; 95% CI, 2.38-20.82 months for OS; 6.57 months; 95% CI, 0.97-12.16 months for RFS) (log-rank *P* <.001 for OS and log-rank *P* <.001 for RFS). When the TIL density was stratified, the OS of patients in the group with an intratumoral TIL density in the highest quartile (median, 52.47 months; 95% CI, 41.98-62.96 months) was significantly longer than that of patients in the other groups (median, 32.83 months; 95% CI, 28.44-37.23 months; *P* = .004). Additionally, as shown in Figure 2C and D, the RFS of patients in the group with an intratumoral TIL density in the highest quartile (median, 21.67 months; 95% CI, 14.43-28.91 months) was significantly longer than that of patients in the other groups (median, 13.55 months; 95% CI, 11.39-15.71 months; *P* = .02). However, differences in survival outcomes were not observed between the stromal TIL density groups (eFigure 2 in Supplement 1). The results of the multivariable Cox proportional hazard models are shown in Table 2. IP was significantly and consistently associated with both OS and RFS. According to this multivariable model, patients with IDP (HR, 5.02; 95% CI, 2.22-11.38; *P* <.001) had the worst prognosis for OS, followed by patients with IEP (HR, 2.41; 95% CI, 1.30-4.50; *P* <.001). Similarly, patients with IDP (HR, 4.12; 95% CI, 1.85-9.17; *P* <.001) had the worst prognosis for RFS, followed by patients with IEP (HR, 2.60; 95% CI, 1.43-4.70; *P* <.001). These results were consistent in the sensitivity analysis model.

**Table 2.** Cox Regression Analysis of Overall Survival (OS) or Recurrence-Free Survival (RFS) With Prognostic Factors

Prognostic factor	Model 1^a^				Model 2^b^			
	OS		RFS		OS		RFS	
	HR (95% CI)	*P* value	HR (95% CI)	*P* value	HR (95% CI)	*P* value	HR (95% CI)	*P* value
IP								
IIP	1 [Reference]	NA	1 [Reference]	NA	1 [Reference]	NA	1 [Reference]	NA
IEP	2.41 (1.30-4.50)	.006	2.60 (1.43-4.70)	.002	2.31 (1.25-4.28)	.008	2.57 (1.42-4.64)	.002
IDP	5.02 (2.22-11.38)	<.001	4.12 (1.85-9.17)	<.001	5.43 (2.40-12.28)	<.001	4.34 (1.96-9.63)	<.001
Age, y	1.04 (1.022-1.057)	<.001	1.025 (1.01-1.040)	.002	1.05 (1.03-1.06)	<.001	1.03 (1.02-1.05)	<.001
Sex								
Female	1.01 (0.75-1.35)	.97	1.02 (0.77-1.34)	.91	1.00 (0.75-1.35)	.99	1.01 (0.77-1.33)	.96
Male	1 [Reference]	NA	1 [Reference]	NA	1 [Reference]	NA	1 [Reference]	NA
Stage								
I	1 [Reference]	NA	1 [Reference]	NA	1 [Reference]	NA	1 [Reference]	NA
II	2.21 (1.58-3.08)	<.001	1.94 (1.43-2.63)	<.001	2.17 (1.55-3.02)	<.001	1.90 (1.40-2.58)	<.001
III	3.68 (2.33-5.80)	<.001	3.04 (1.99-4.66)	<.001	4.98 (3.24-7.64)	<.001	3.88 (2.57-5.85)	<.001
Adjuvant therapy								
No	1 [Reference]	NA	1 [Reference]	NA	NA	NA	NA	NA
Yes	0.50 (0.35-0.71)	<001	0.48 (0.35-0.67)	<.001	NA	NA	NA	NA

Abbreviations: HR, hazard ratio; IDP, immune-desert immunophenotype; IEP, immune-excluded immunophenotype; IIP, immune-inflamed immunophenotype; IP, immunophenotype; NA, not applicable.

^a^ Model 1: Cox regression with immunophenotype, age, sex, stage, adjuvant treatment.

^b^ Model 2: Cox regression with immunophenotype, age, sex, stage (for sensitivity analysis).

### Impact of Adjuvant Therapy on Survival by IP

Among patients who received adjuvant therapy (n = 238), OS and RFS differed significantly by IP. The IIP group had the most favorable prognosis, with median OS and RFS not reached (eFigure 3A and B in Supplement 1). The IEP group had a median OS of 44.05 months (95% CI, 37.26-50.84 months; HR, 1.45; 95% CI, 0.78-2.69; *P* = .004) and a median RFS of 18.27 months (95% CI, 13.45-23.09 months; HR, 1.70; 95% CI, 0.94-3.08; *P* = .02), whereas the IDP group had the worst outcomes (median OS, 11.60 months; 95% CI, 0-23.51 months; HR, 3.96; 95% CI, 1.64-9.59; *P* = .004; median RFS, 6.57 months; 95% CI, 1.79-11.35 months; HR, 3.33; 95% CI, 1.40-7.92; *P* = .02). Among patients who did not receive adjuvant therapy (n = 66), OS and RFS also varied significantly by IP (eFigure 3 C and D in Supplement 1). The IEP group had a median OS of 15.19 months (95% CI, 12.66-20.22 months; HR, 11.14; 95% CI, 4.15-23.26; *P* = .01) and a median RFS of 6.32 months (95% CI, 3.60-9.04 months; HR, 11.16; 95% CI, 1.42-24.90; *P* = .02), whereas the IDP group had an OS of 14.26 months (95% CI, 5.87-indeterminate months; HR, 11.88; 95% CI, 1.19-114.11; *P* = .01) and an RFS of 6.59 months (95% CI, 1.07-indeterminate months; HR, 12.56; 95% CI, 1.08-188.50; *P* = .02). These findings suggest that IEP patients may benefit from adjuvant therapy.

Further analysis of adjuvant treatment modalities revealed that patients with IIP who received chemotherapy had superior survival, with median OS and RFS not reached **(**eFigure 4A and B in Supplement 1). However, patients with IIP treated with radiotherapy had worse outcomes (median OS, 20.45 months; 95% CI, 8.72-32.18 months; HR, 6.11; 1.82-20.51; *P* <.001; median RFS, 13.61 months (95% CI, 6.09-21.13 months; HR, 3.65; 95% CI, 1.14-11.72; *P* = .02). No significant differences in outcomes were observed between adjuvant treatments in patients with IEP and IDP (eFigure 4C-F in Supplement 1).

### Pathological Stage and IP as Prognostic Factors

**Figure 3.**
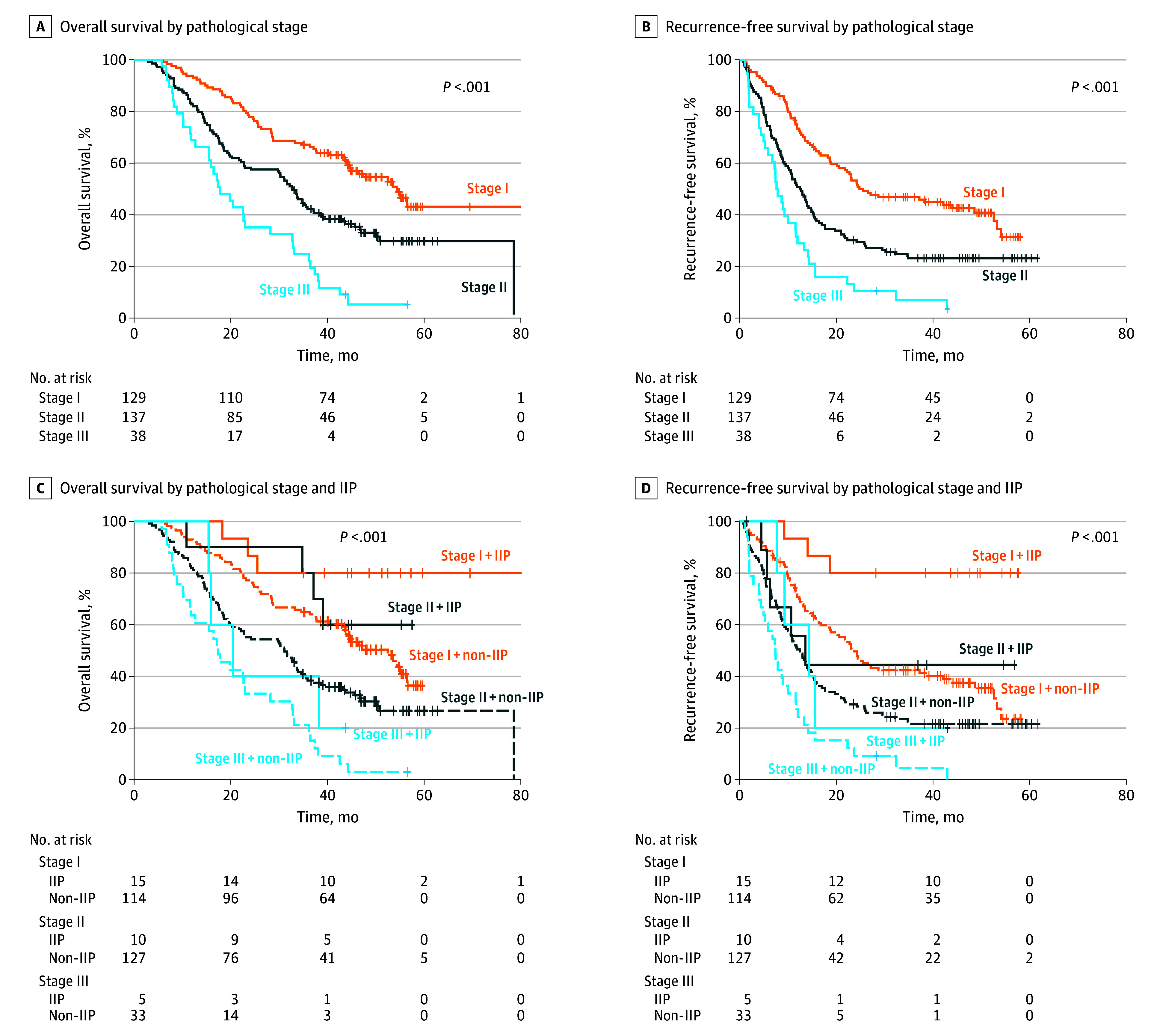
Survival Outcome Prediction According to Tumor Stage and Immune Phenotype A, Overall survival according to pathological stage. B, Recurrence-free survival according to the pathological stage. C, Overall survival and recurrence-free survival (D) according to pathologic stage stratified by immune-inflamed phenotype (IIP) or non-IIP (immune-excluded phenotype + immune-desert phenotype).

Pathological stage effectively predicted prognosis in Kaplan-Meier and Cox proportional hazard analysis (Figure 3A and B). Median OS was longest in stage I (54.43 months), followed by stage II (32.61 months; HR, 1.90; 95% CI, 1.37-2.63), and shortest in stage III (17.33 months; HR, 4.03; 95% CI, 2.65-6.12; *P* <.001). Similarly, median RFS decreased from stage I (25.55 months) to stage II (12.69 months; HR, 1.82; 95% CI, 1.35-2.45) and stage III (7.66 months; HR, 3.40; 95% CI, 2.27-5.09).

When we further analyzed OS and RFS according to pathologic stage combined with IP stratification, IIP had superior OS and RFS across all stages (Figure 3C and D). Using stage I and IIP as a reference, patients with stage I and non-IIP had shorter OS (52.47 months; HR, 3.25; 95% CI, 1.02-10.42), whereas those with stage II and IIP showed prolonged OS (not reached; HR, 2.35; 95% CI, 0.53-10.55). Stage II and non-IIP had a median OS of 31.0 months (HR, 5.93; 95% CI, 1.87-18.81), and stage III and IIP had 20.45 months (HR, 7.76; 95% CI, 1.73-34.89). The worst OS was observed in stage III and non-IIP (17.33 months; HR, 12.71; 95% CI, 3.86-41.80). Remarkably, patients with stage II and IIP tended to have longer OS than those with stage I and non-IIP.

RFS followed a similar trend, with stage I and IIP as the reference. Stage I and non-IIP had a median RFS of 23.15 months (HR, 4.44; 95% CI, 1.40-14.09), whereas stage II and IIP had 13.61 months (HR, 4.23; 95% CI, 1.01-17.72). Stage II and non-IIP (12.50 months; HR, 7.38; 95% CI, 2.34-23.40) and stage III and IIP (14.40 months; HR, 7.18; 95% CI, 1.60-32.16) had worse outcomes, with stage III and non-IIP showing the shortest RFS (7.40 months; HR, 14.98; 95% CI, 4.57-49.17).

## Discussion

This cohort study explored the prognostic role of IP, identified via an AI-powered spatial TIL analyzer, in resected PDAC. IP remained an independent predictor of RFS and OS, even after adjusting for clinicopathologic factors. This was the first study, to our knowledge, to apply AI-driven TIL analysis to R0 resected PDAC, demonstrating its potential to enhance prognostic accuracy while reducing manual effort. Our findings suggest that integrating IP into the TNM staging system could improve outcome predictions. Although determining the optimal TIL density is challenging, higher intratumoral TIL densities correlated with better survival, emphasizing the prognostic value of IP in PDAC.

TILs are valuable prognostic markers across solid tumors, including PDAC.^6,7,8,9,10,13,14,15,16,17^ However, heterogeneity in prior studies, such as varied tissue assessment methods, TIL evaluation criteria, cutoff values, and reliance on manual inspection, has hindered their clinical adoption. Manual TIL evaluation is labor intensive, subject to interobserver and intraobserver variability, and may require immunohistochemistry. In contrast, AI-based analysis ensures consistency, reproducibility, and reduces reliance on additional resources by solely relying on H&E-stained slides. This consistency is particularly beneficial in PDAC, where intratumoral TIL densities are low and small observer variations may lead to exaggerated interpretations. Although our model does not distinguish lymphocyte subtypes such as regulatory T lymphocytes or myeloid-derived suppressor cells, it provides reliable prognostic value using only standard H&E slides without the need for immunohistochemistry. We have also reported that there was a strong positive correlation of TIL densities between this AI model and the pathologists’ TIL evaluation according to the International TILs Working Group guideline,^18^ reinforcing the reliability of AI-driven classification. In our study, the automated classification of IPs and calculation of higher intratumoral TIL levels based on simple H&E-stained slides demonstrated robust performance, emphasizing the practical importance of spatial TIL distribution patterns. Advances in single-cell trajectory analyses have provided deeper insights into the TIL landscape in PDAC.^19^ Despite the presence of tumor-reactive TILs, the unexplained lack of response to immunotherapy in patients with PDAC raises important questions about the TME and its influence on TIL distribution and function. Although our approach did not quantify single-cell proximity, the differential prognostic value of intratumoral vs stromal TILs highlights the clinical relevance of spatial immune localization.^20^ Future research should refine TIL subset distributions for more precise prognosis prediction while simplifying methodologies to enhance clinical applicability. Consequently, these biomarkers have the potential for widespread use in routine clinical practice, and this potential is further amplified considering the current advancements in T-cell priming, T-cell expansion, and T-cell trafficking involved in a variety of therapeutic measures in the era of immuno-oncology drugs.^21,22,23,24^ Practical application requires determining optimal cutoffs for IP classification. Although non–small cell lung cancer studies used a 33.3% threshold and 100/mm^2^ density,^11^ we adopted a 20% threshold or 200/mm^2^ density to account for lower immune infiltration in PDAC. Further research is needed to establish optimal cutoffs across different types of cancers. Additionally, it is crucial to determine and extend the tailored treatment strategy based on the IP of the tumor to broader patient cohorts, considering the patient’s disease status and history of prior treatments, rather than limiting the approach to patients who undergo complete resection without prior anticancer treatment as in this study.

### Limitations

This study has several limitations. First, to reduce the influence of other significant prognostic factors, this study was designed to include patients who underwent R0 resection without neoadjuvant therapy and who did not have distant metastasis at diagnosis. However, the corresponding tumors are not commonly encountered in real-world practice and account for only 10% to 20% of all PDAC cases. Therefore, future studies should include patients who have received neoadjuvant therapy before surgical resection or those with unresectable disease who undergo surgical resection following palliative treatment. Additionally, the patients in this study were treated in a manner that might be considered outdated by current standards like receiving adjuvant treatment with gemcitabine alone, and this approach may introduce selection bias. Additionally, we acknowledge that HRs are subject to inherent selection bias, as they can be influenced by baseline characteristics and time-dependent variations in risk. This built-in selection bias may limit the causal interpretation of HRs within the retrospective cohort study design.^25^ Next, we identified TILs in surgically resected specimens and selected a representative slide with the largest tumor area for analysis. Although we thoroughly investigated all aspects of the selected sections, importantly, this approach may not fully capture the entire tumor-level IP due to the inherent limitations of this methodology. Future efforts should identify the optimal selection of representative tumor slides or explore feasible methods to assess whole-tumor–level IP. Furthermore, classifying IP with biopsy tissue specimens is an essential step that will unlock further utilization of this technology, providing deeper insights into IP with unresectable PDACs and dynamic changes in TILs in response to treatment. Last, we did not differentiate lymphocyte subtypes with this AI model because Lunit SCOPE was developed to automatically classify IPs and calculate TILs based on simple H&E-stained slides for practical use. We used TCGA data to characterize IPs with immune cell types and showed that IIP was significantly related to higher CD8+ T cell fraction and immune cytolytic activity. The determination and evaluation of each immune cell type could be important to understand the tumor microenvironment and to predict the prognosis in PDAC more accurately, so we are developing an algorithm to further distinguish cell types in H&E-stained images.

## Conclusions

Results of this cohort study suggest that the AI-powered TIL analyzer markedly condensed the labor-intensive process of TIL assessment and demonstrated the ability to evaluate TIL status to predict survival outcomes in patients with PDAC. Importantly, the immunophenotype can be one of the most important biomarkers for OS and RFS, complementing the pathologic TNM staging system for resected PDAC. Notably, a greater density of intratumoral TILs was associated with improved survival outcomes. Future research should aim to evaluate the utility of this technology in patients receiving anticancer therapy, including those with advanced PDAC, and extend its application to biopsy specimens, which could enable preoperative classification of the immunophenotype and broader clinical integration. Additionally, it is essential to validate the effectiveness of this approach in large-scale human studies to establish its broader applicability and reliability in clinical settings. Furthermore, immunophenotype classification strategies, including cutoff thresholds, the use of categorical vs continuous scoring, and the potential integration with molecular subtypes, may require refinement as more PDAC-specific data become available.

## Notes

Supplement 1. **eFigure 1.** Overview of the Study **eFigure 2.** Comparison of Survival Outcomes According to Stromal Tumor-Infiltrating Lymphocyte **eFigure 3.** Kaplan-Meier Analysis for the Comparison of Survival Outcomes in Patients With or Without Adjuvant Therapy **eFigure 4.** Kaplan-Meier Analysis for the Comparison of Survival Outcomes in Patients According to the Type of Adjuvant Therapy **eTable 1.** Baseline Characteristics by Immune Phenotype **eTable 2.** Distribution of Intratumoral or Stromal Tumor-Infiltrating Lymphocytes by Clinicopathologic Features and Immunophenotype
eMethods.

eReferences.

Supplement 2.
Data Sharing Statement.
