# Supplementary material for: Artificial Intelligence–Powered Spatial Analysis of Immune Phenotypes in Resected Pancreatic Cancer
Source: JAMA Surg. 2025 Jun 25;160(8):884–92. doi: 10.1001/jamasurg.2025.1999 (PMC12199178; doi:10.1001/jamasurg.2025.1999)
Supplement: Supplement 1. — eFigure 1. Overview of the Study eFigure 2. Comparison of Survival Outcomes According to Stromal Tumor-Infiltrating Lymphocyte eFigure 3. Kaplan-Meier Analysis for the Comparison of Survival Outcomes in Patients With or Without Adjuvant Therapy eFigure 4. Kaplan-Meier Analysis for the Comparison of Survival Outcomes in Patients According to the Type of Adjuvant Therapy eTable 1. Baseline Characteristics by Immune Phenotype eTable 2. Distribution of Intratumoral or Stromal Tumor-Infiltrating Lymphocytes by Clinicopathologic Features and Immunophenotype eMethods. eReferences. [file jamasurg-e251999-s001.pdf]

Supplementary Online Content

Kim H, Choi JH, Lim Y, et al. Artificial intelligence–powered spatial analysis of immune phenotypes in resected pancreatic cancer. *JAMA Surg*. Published online June 25, 2025. doi:10.1001/jamasurg.2025.1999

eFigure 1. Overview of the Study

eFigure 2. Comparison of Survival Outcomes According to Stromal Tumor-Infiltrating Lymphocyte

eFigure 3. Kaplan-Meier Analysis for the Comparison of Survival Outcomes in Patients With or Without Adjuvant Therapy

eFigure 4. Kaplan-Meier Analysis for the Comparison of Survival Outcomes in Patients According to the Type of Adjuvant Therapy

eTable 1. Baseline Characteristics by Immune Phenotype

eTable 2. Distribution of Intratumoral or Stromal Tumor-Infiltrating Lymphocytes by Clinicopathologic Features and Immunophenotype

eMethods.

eReferences.

This supplementary material has been provided by the authors to give readers additional information about their work.

© 2025 Kim H et al. *JAMA Surgery.*

eFigure 1. Overview of the study

(A) Schematic workflow of the study. (B) Flowchart of selection of study patients. IIP, immune inflamed phenotype; IEP, immune excluded phenotype; IDP, immune desert phenotype.

(A)

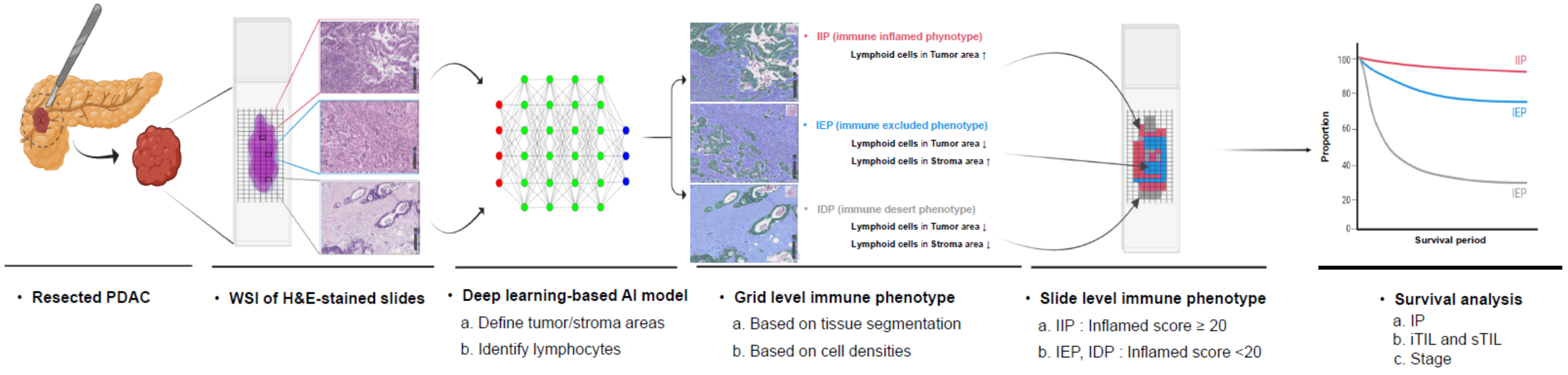

(B)

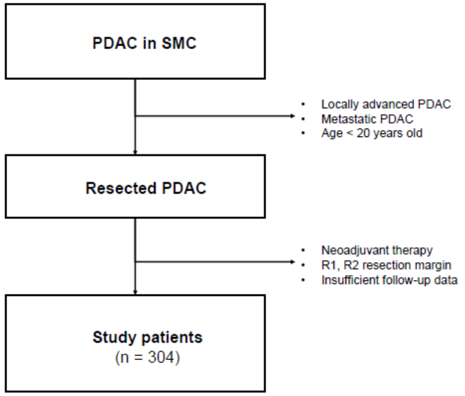

© 2025 Kim H et al. *JAMA Surgery.*

Figure 2. Comparison of survival outcomes according to stromal tumor-infiltrating lymphocytes

(A) overall survival (B) recurrence-free survival according to stromal tumor-infiltrating lymphocyte (sTIL) density

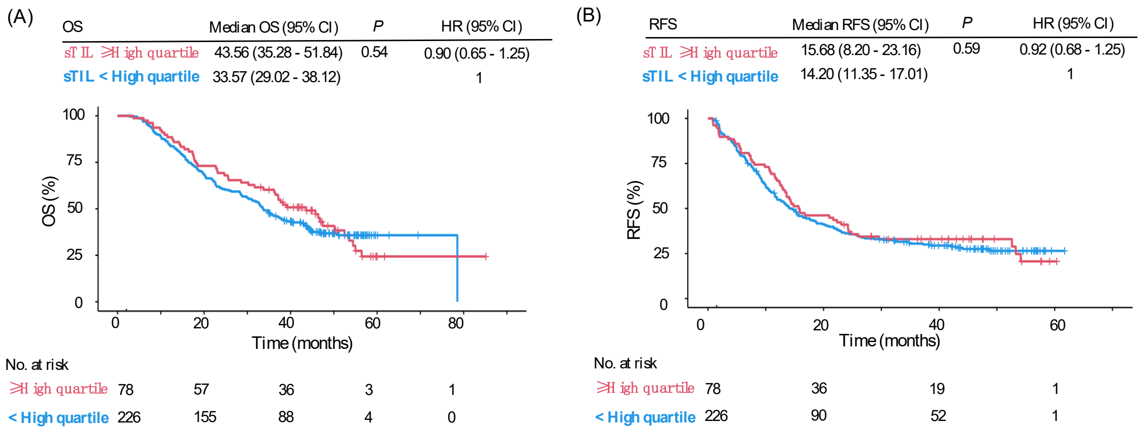

© 2025 Kim H et al. *JAMA Surgery*.

eFigure 3. Kaplan-Meier analysis for the comparison of survival outcomes in patients with or without adjuvant therapy.

(A) OS in patients who received adjuvant therapy, according to the IP. (B) RFS in patients who received adjuvant therapy, according to the IP. (C) OS in patients who did not receive adjuvant therapy, according to the IP. (D) RFS in patients who did not receive adjuvant therapy, according to the IP; IIP, immune inflamed phenotype; IEP, immune excluded phenotype; IDP, immune desert phenotype.

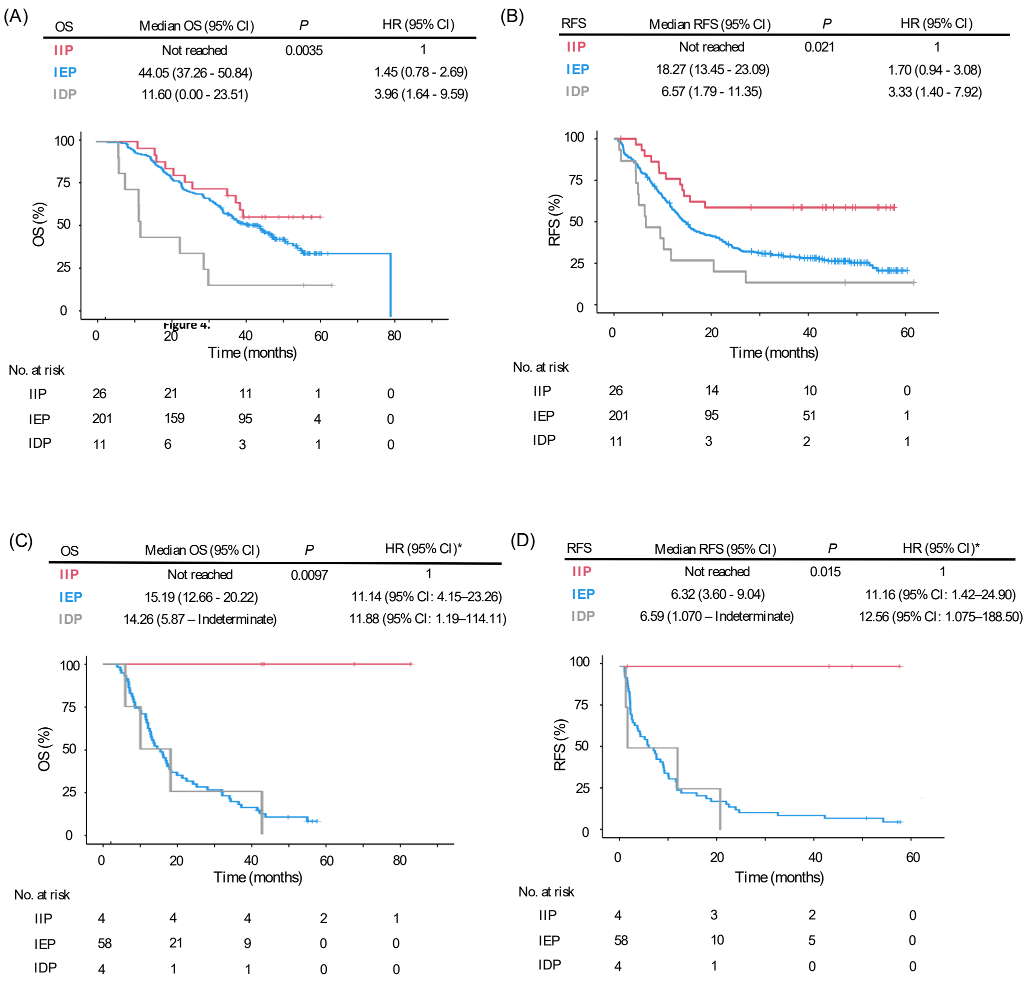

*These HRs were calculated using Ridge-based Penalized Cox Regression

© 2025 Kim H et al. *JAMA Surgery.*

eFigure 4. Kaplan-Meier analysis for the comparison of survival outcomes in patients according to the type of adjuvant therapy.

(A) OS in the patients with IIP. (B) RFS in the patients with IIP. (C) OS in the patients with IEP. (D) RFS in the patients with IEP. (E) OS in the patients with IDP. (F) RFS in the patients with IDP.

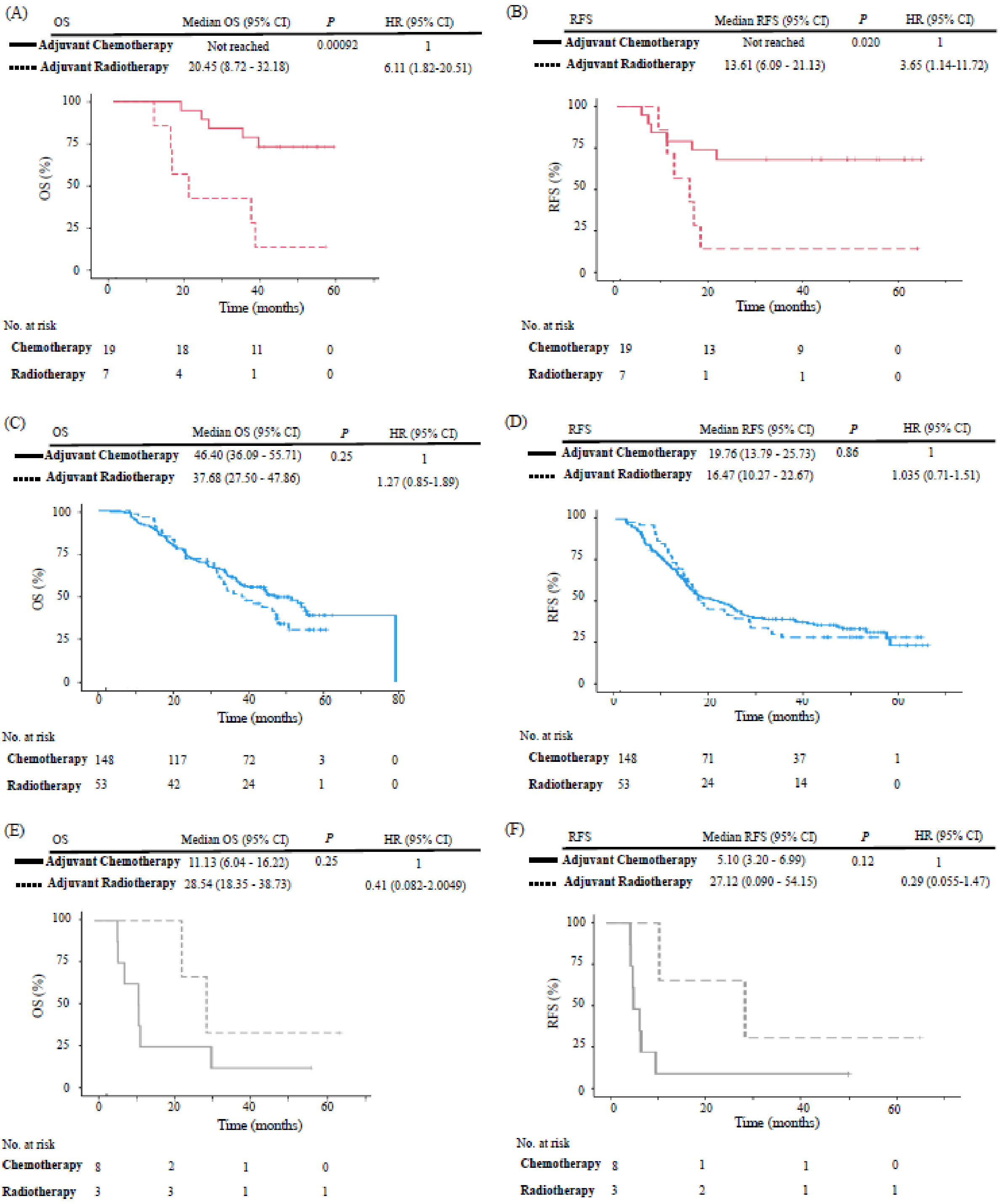

© 2025 Kim H et al. *JAMA Surgery*.

eTable 1. Baseline Characteristics by Immune Phenotype

		IIP (n=30)	IEP (n=259)	IDP (n=15)	*P*
**Age (years)**	mean±SD	64.3±13.1	66.9±8.9	71.5±8.33	0.069
**Sex**	Male	15	147	9	0.745
	Female	15	112	6	
**Pathologic Stage (AJCC 8th)**	I	15	110	4	0.487
	II	10	118	9	
	III	5	31	2	
**Lymphovascular invasion**	Positive	10	116	8	0.222
	Negative	20	138	7	
	NA	0	5	0	
**Perineural invasion**	Positive	24	216	12	0.804
	Negative	6	38	3	
	NA	0	5	0	
**Differentiation**	WD, MD	20	201	9	0.211
	PD, UD	10	53	6	
	NA	0	5	0	
**Adjuvant therapy**	Yes	26	201	11	0.466
	No	4	58	4	

AJCC, American Joint Committee on Cancer; NA, not available; WD, well differentiated; MD, moderately differentiated; PD, poorly differentiated; UD, undifferentiated.

© 2025 Kim H et al. *JAMA Surgery.*

eTable 2. Distribution of intratumoral or stromal tumor-infiltrating lymphocytes by clinicopathologic features and immunophenotype

Clinicopathologic features		TIL density (mean, /mm^2)			
		iTIL (/mm^2)	*P*	sTIL(/mm^2)	*P*
**Entire study patients**		100.64		734.88	
**Immune Phenotypes**	**IIP**	286.04	<0.001	1069.00	<0.001
	**IEP**	81.11		723.99	
	**IDP**	67.08		254.73	
**Age (years)**	**< 67**	108.35	0.13	787.31	0.042
	**≥ 67**	93.800		688.32	
**Pathologic Stage (AJCC 8th)**	**I**	101.18	0.94	697.10	0.28
	**II**	99.18		747.56	
	**III**	104.32		817.48	
**Pathologic T stage**	**T1**	114.76	0.22	792.65	0.070
	**T2**	95.23		739.11	
	**T3**	109.40		570.82	
**Pathologic N stage**	**N0**	105.5	0.57	686.07	0.13
	**N1**	94.7		760.10	
	**N2**	103.0		825.54	
**Lymphovascular invasion**	**No**	109.76	0.089	684.28	0.07
	**Yes**	93.24		773.34	
**Perineural invasion**	**No**	116.32	0.33	756.82	0.64
	**Yes**	98.46		725.19	
**Differentiation**	**WD, MD**	97.05	0.19	745.45	0.36
	**PD, UD**	115.38		690.87	

TIL, tumor infiltrating lymphocytes; iTIL, intratumoral tumor infiltrating lymphocytes; sTIL, stromal tumor infiltrating lymphocytes; IP, immunophenotype; IIP, immune-inflamed immunophenotype; IEP, immune-excluded immunophenotype; IDP, immune-desert immunophenotype; AJCC, American Joint Committee on Cancer; WD, well-differentiated; MD, moderately differentiated; PD, poorly differentiated; UD, undifferentiated

© 2025 Kim H et al. *JAMA Surgery.*

eMethods

Spatial TIL analysis via an AI-powered WSI analyzer, Lunit SCOPE IO

Surgical specimen sections were stained with H&E and scanned at 40x magnification using an Aperio ScanScope® AT2 System (Leica Microsystems) for further AI-powered WSI analysis. Lunit SCOPE IO (Lunit, Inc., Seoul, Korea) is a deep learning-based spatial TIL analyzer composed of two complementary AI models for cell detection and tissue segmentation. As previously described, the proposed method is based on the DeepLabV3+ convolutional neural network architecture, with a ResNet-34 backbone serving as a feature extractor.[1-5] The models were developed and trained with patches extracted from WSIs, annotated and segmented by board-certified pathologists and updated from a previous version using 13.5 x 10^9 μm^2 tissue regions and 6.2 x 10^5 TILs on 17,292 H&E-stained WSIs from 17 tumor types, including pancreatic cancer.[1,6] The cell detection AI model identifies the location of tumor cells and lymphocytes. In contrast, the tissue segmentation AI model determines whether a pixel belongs to a CA, peritumoral stroma, or a nontumor background region. The performance of the version of the model used in this study, assessed using the tuning dataset, yielded a mean intersection over union (mIoU) of 0.74 for segmentation and an mF1 score of 0.71 for cell detection. The model's performance in the pancreatic cancer subset displayed a mIoU of 0.61 for segmentation and an mF1 of 0.71 for cell detection.

The workflow is as follows: First, the system recognized TILs within spatial segmentation contexts from H&E-stained WSIs. TILs were quantified to calculate one mm^2-sized grid-level TIL density in two areas of interest: 1) the iTIL density, defined as the TIL count within the CA in a grid, and 2) the stromal TIL (sTIL) density, defined as the TIL count within the CS in a grid. The spatial TIL density information was used to classify the IP of each grid: 1) immune-inflamed IP (IIP), defined as an iTIL density $\geq 200/mm^2$; 2) immune-excluded phenotype (IEP), defined as an iTIL $< 200/mm^2$ and an sTIL $\geq 200/mm^2$; and 3) immune-desert phenotype (IDP), defined as both an iTIL and an sTIL $< 200/mm^2$.[6] The WSI-level inflamed score (IS), immune-excluded score, and immune-desert score were calculated by dividing the number of grids classified as each respective IP by the number of all grids analyzed. Finally, slide-level IPs were defined using the predefined cancer-type agnostic IS threshold, which was determined before this study to distinguish patients who exhibited a high T-cell inflamed gene expression profile as represented by the interferon-gamma (*IFNG*)-responsive gene signature[7] with the best sensitivity and specificity.

TCGA data analysis

Among 183 RNA sequencing data in The Cancer Genome Atlas (TCGA)-pancreatic adenocarcinoma (PAAD) cohort, 172 RNA sequencing data with qualified H&E WSI were used for analyzing each subgroup's immune cell composition and immune cytotoxic activity. CIBERSORTx was used for the immune cell composition analysis,[8] and the input was the TPM (transcripts per million)-normalized expression of the TCGA PAAD cohort. According to the instructions of CIBERSORTx, we selected the LM22 (22 immune cell types) signature matrix, B-mode batch correction batch correction, and 1000 permutations. The significance of CIBERSORTx among the three IPs was evaluated using both parametric and non-parametric tests. We tested for the normality of distribution and homogeneity of variance across IPs. If both normality and homogeneity of variance were met, we conducted an ANOVA test. If normality was met but the homogeneity of variance was not, we used Welch's ANOVA. If normality was not met in any group, we performed the non-parametric Kruskal-Wallis H test. For post hoc tests, we applied the Games-Howell test when significance was found in Welch's ANOVA, and Dunn's test was applied when significance was found in the Kruskal-Wallis test. The cytolytic activity (CYT) score[9] and IFNG score[7] were used to compare the immune cytotoxic activity of each TIL quantification subtype, calculated as the mean of log2 transformation of TPM. The CYT score was calculated for both *GZMA* and *PRF1*, and the IFNG score was calculated for the genes *IDO1, CXCL10, CXCL9, HLA-DRA, STAT1,* and *IFNG.* Statistical significance was tested with one-way analysis of variance (ANOVA) followed by Fisher's least significant difference post hoc test, with significance set at 0.05.

© 2025 Kim H et al. *JAMA Surgery.*

Statistical analysis

Differences in means for continuous variables between groups were compared using the Wilcoxon rank-sum or Kruskal–Wallis test. The Kaplan–Meier method was used to estimate recurrence-free survival (RFS) and OS. Survival functions and median survival times were estimated using the Kaplan-Meier method, and the 95% confidence intervals (CIs) for the median survival times were calculated using Greenwood's formula. The start time for OS was defined as the date of diagnosis, while for RFS, the start time was the date of surgery. Survival outcomes were measured from these points until the occurrence of specified events. The event for OS was defined as death from any cause up to the last follow-up, while the events for RFS were defined as either disease recurrence or death from any cause up to the last follow-up. Patients who did not experience an event were treated as censored data. Medical records of the study patients were reviewed through August 2023. The log-rank test was used to assess differences between the groups regarding RFS or OS. Univariable and multivariable comparisons were performed using the Cox proportional hazards model to adjust for confounders, ensuring a robust estimation of the prognostic value of the IP with the package "*survival*" of R. If it was challenging to calculate the hazard ratio using Cox regression, particularly in cases of complete separation or rare events, a ridge-based penalized Cox regression model and we employed bootstrapping with a bias-corrected and accelerated method to estimate the 95% confidence interval of the hazard ratio, utilizing the "*glmnet*" package of R. A Directed Acyclic Graph combining causal and associative components with the disjunctive cause criterion was used to identify the minimally sufficient set of confounders for the multivariable model based on both the study results and literature evidence in PDAC with the package "*dagitty*" of R.[10-12] For the identified confounding variables related to IP, the proportional hazards assumption was evaluated using the Schoenfeld Residuals Test. Sensitivity analyses were conducted for variables where a potential violation of the proportional hazards assumption was detected. Additionally, Martingale Residuals were plotted to evaluate the linearity of quantitative predictors. Two-sided p values were reported, and p values less than 0.05 indicated statistical significance. The statistical analysis used 'R' version 4.2.2 and SPSS version 23.0.

© 2025 Kim H et al. *JAMA Surgery.*

eReferences

1. Park S, Ock CY, Kim H, et al. Artificial Intelligence-Powered Spatial Analysis of Tumor-Infiltrating Lymphocytes as Complementary Biomarker for Immune Checkpoint Inhibition in Non-Small-Cell Lung Cancer. *J Clin Oncol.* 2022;40(17):1916-1928.
2. Jung HA, Park KU, Cho S, et al. A Phase II Study of Nivolumab plus Gemcitabine in Patients with Recurrent or Metastatic Nasopharyngeal Carcinoma (KCSG HN17-11). *Clin Cancer Res.* 2022;28(19):4240-4247.
3. Lim Y, Choi S, Oh HJ, et al. Artificial intelligence-powered spatial analysis of tumor-infiltrating lymphocytes for prediction of prognosis in resected colon cancer. *NPJ Precis Oncol.* 2023;7(1):124.
4. He K, Zhang X, Ren S, Sun J. Deep Residual Learning for Image Recognition. Paper presented at: 2016 IEEE Conference on Computer Vision and Pattern Recognition (CVPR); 27-30 June 2016, 2016.
5. Chen L-C, Papandreou G, Schroff F, Adam H. Rethinking Atrous Convolution for Semantic Image Segmentation. *ArXiv.* 2017;abs/1706.05587.
6. Shen J, Choi YL, Lee T, et al. Inflamed immune phenotype predicts favorable clinical outcomes of immune checkpoint inhibitor therapy across multiple cancer types. *J Immunother Cancer.* 2024;12(2).
7. Ayers M, Lunceford J, Nebozhyn M, et al. IFN-γ-related mRNA profile predicts clinical response to PD-1 blockade. *J Clin Invest.* 2017;127(8):2930-2940.
8. Newman AM, Steen CB, Liu CL, et al. Determining cell type abundance and expression from bulk tissues with digital cytometry. *Nat Biotechnol.* 2019;37(7):773-782.
9. Rooney MS, Shukla SA, Wu CJ, Getz G, Hacohen N. Molecular and genetic properties of tumors associated with local immune cytolytic activity. *Cell.* 2015;160(1-2):48-61.
10. Greenland S, Pearl J, Robins JM. Causal diagrams for epidemiologic research. *Epidemiology.* 1999;10(1):37-48.
11. Mickey RM, Greenland S. The impact of confounder selection criteria on effect estimation. *Am J Epidemiol.* 1989;129(1):125-137.
12. Maldonado G, Greenland S. Simulation study of confounder-selection strategies. *Am J Epidemiol.* 1993;138(11):923-936.

© 2025 Kim H et al. *JAMA Surgery.*
