# Supplementary material for: Artificial Intelligence–Powered Spatial Analysis of Immune Phenotypes in Resected Pancreatic Cancer
Source: JAMA Surg. 2025 Jun 25;160(8):884–92. doi: 10.1001/jamasurg.2025.1999 (PMC12199178; doi:10.1001/jamasurg.2025.1999)
Supplement: Supplement 2. — Data Sharing Statement. [file jamasurg-e251999-s002.pdf]

Data Sharing Statement

Kim. Artificial Intelligence–Powered Spatial Analysis of Immune Phenotypes in Resected Pancreatic Cancer. *JAMA Surg*. Published June 25, 2025. doi:10.1001/jamasurg.2025.1999

Data
Data available: No

Additional Information
Explanation for why data not available: Data may be made available upon reasonable request to the corresponding author.
